# Molecular regulation and therapeutic targeting of *MYCN* in neuroblastoma: a comprehensive review

**DOI:** 10.3389/fcell.2025.1683331

**Published:** 2025-10-30

**Authors:** Yi Chen, Huixian Yang, Lirong Xiao, Naihong Yan, Ming Zhang

**Affiliations:** ^1^ Department of Ophthalmology, West China Hospital, Sichuan University, Chengdu, Sichuan, China; ^2^ Department of Ophthalmology and Research Laboratory of Ophthalmology, West China Hospital, Sichuan University, Chengdu, Sichuan, China

**Keywords:** *MYCN* amplification, N-myc, *MYCN* protein, neuroblastoma, epigenetic regulation, PROTAC, noncoding RNA, metabolic reprogramming

## Abstract

*MYCN* amplification defines a highly aggressive subtype of neuroblastoma and is strongly associated with poor clinical outcomes. Due to the intrinsically disordered structure of the N-Myc protein, it remains largely undruggable. This review provides a comprehensive summary of the molecular regulatory network surrounding *MYCN*, including upstream pathways, key cofactors, and downstream effectors involved in cell cycle control, metabolic reprogramming, and ferroptosis. We further discuss the roles of epigenetic modulators, noncoding RNAs, and positive feedback loops in sustaining *MYCN*-driven oncogenic programs. Emerging therapeutic strategies such as PROTACs, metabolic inhibitors, immune-based approaches, and RNA-targeting technologies offer promising alternatives to direct *MYCN* inhibition. This review aims to provide a theoretical foundation for future development of precise and effective therapies targeting *MYCN*-amplified neuroblastoma.

## 1 Introduction

The *V-myc avian myelocytomatosis viral oncogene neuroblastoma-derived homolog* (*MYCN*) gene is a member of the *MYC* oncogene family and is located in the short arm of the human chromosome 2, 2p24.3 region (Nishio et al., 2024). *MYCN* is highly expressed in many cancers, including neuroblastoma (NB) (Huang and Weiss, 2013), medulloblastoma (Korshunov et al., 2012), cervical cancer (García et al., 2024), and non-small cell lung cancer (Dietzsch et al., 1994). Approximately 20% of NBs exhibit *MYCN* amplification (*MYCN*-amplified neuroblastoma, MNA-NB) (Huang and Weiss, 2013). *MYCN* promotes NB growth mainly by forming a complex with MDM2 and inhibiting the action of the p53 protein (Slack et al., 2005). *MYCN* amplification is positively correlated with poor prognosis in NB patients and negatively correlated with overall survival (OS) (Valentijn et al., 2012; Campbell et al., 2019). *MYCN* encodes the N-Myc protein that lacks the clear hydrophobic pocket that small molecules usually target and is considered undruggable. Currently, there are still no drugs that directly target N-Myc (Bushweller, 2019; Wolpaw et al., 2021).

This review systematically discusses the molecular regulatory network related to *MYCN* in NB and summarizes the indirect intervention strategies targeting the *MYCN* gene and its products from the current research on MNA-NB. Through a comprehensive analysis of the biological functions and pathogenic mechanisms of *MYCN*, this review aims to provide a theoretical basis for the development of new therapeutic strategies with *MYCN* amplification as a therapeutic target, especially for neuroblastoma.

## 2 Current treatment options for MNA-NB

NB shows characteristic molecular alterations that shape its biology and clinical outcome. The most frequent events include *MYCN* amplification, *ALK* mutations or amplification, and alterations of *ATRX* and *TERT* (Pugh et al., 2013; Pinto et al., 2015). Beyond these drivers, NB cells are increasingly recognized to depend on aberrant RNA splicing, with dysregulated splicing regulators (e.g., *SF3B1*, *HNRNPA1*, *PTBP1*) supporting proliferation and survival (Zhang et al., 2016; Bonnal et al., 2020; Shi et al., 2021). Notably, *MYCN* amplification strengthens this reliance, as N-Myc cooperates with spliceosomal machinery and sensitizes NB cells to spliceosome inhibition (Hsu et al., 2015; Kress et al., 2015). These findings indicate that NB pathogenesis involves both canonical genetic alterations and post-transcriptional vulnerabilities that may be therapeutically exploitable. Given its pivotal role in tumor progression, the subsequent discussion will primarily focus on *MYCN* and its associated molecular mechanisms.

All NBs with *MYCN* amplification are considered high risk, regardless of the stage or other risk factors (Irwin et al., 2021). There is currently no treatment targeting *MYCN*. Patients with MNA-NB are treated according to the treatment of high-risk NB, on the basis of the results of phase III trials and open clinical trials. The initial induction therapies include cisplatin, alkylators (Kushner et al., 2004), autologous stem cell collection and transplantation (Park et al., 2019). The efficacy of new regimens such as 131I-MIBG or anti-GD2 (Disialoganglioside GD2) monoclonal antibody therapy is currently being researched in clinical trials (McCluskey et al., 2005; Del Bufalo et al., 2023). The efficacy of induction therapy is limited, and patients with a poor response to treatment receive consolidation therapy (Pinto et al., 2019), using high-dose chemotherapy with autologous stem cell rescue, followed by radiotherapy of primary and metastatic lesions (Zhao et al., 2020). Patients without disease progression after consolidation therapy receive postconsolidation therapy containing the anti-GD2 antibody Dinutuximab and isotretinoin (Yu A. L. et al., 2021). Patients who still progress receive chemoimmunotherapy or participate in clinical trials. Eflornithine [2,5-diamino-2-(difluoromethyl) pentanoic acid hydrochloride hydrate] is an inhibitor of ornithine decarboxylase that has been approved by the FDA for patients who have responded to anti-GD2 immunotherapy (Oesterheld et al., 2024; Nazir et al., 2024; Bagatell et al., 2024).

**FIGURE 1:**
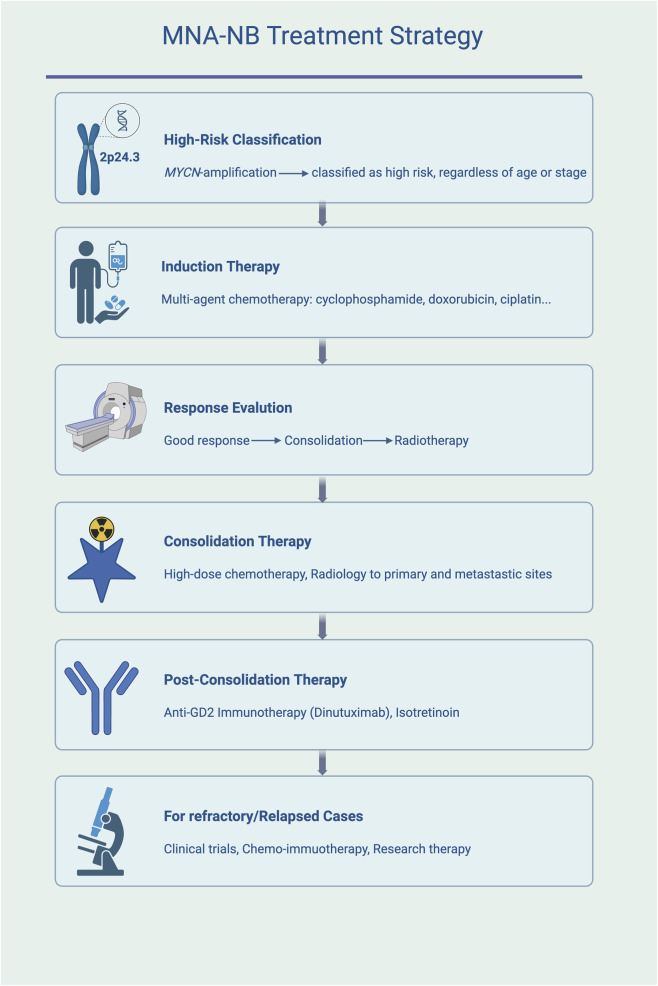
Treatment algorithm for MNA-NB. High-risk classification is assigned to all cases with *MYCN* amplification. Standard management includes induction chemotherapy, consolidation with high-dose chemotherapy and radiotherapy, post-consolidation anti-GD2 immunotherapy and isotretinoin, and experimental approaches for refractory or relapsed disease.

To highlight translational relevance, Table 1 summarizes representative clinical trials in neuroblastoma, including ongoing and completed studies with different treatment strategies. Figure 1 presents the treatment algorithm for MNA-NB (Figure 1; Table 1).

**TABLE 1:** Representative interventional clinical trials in neuroblastoma.

NCT number	Current status	Intervention category	Phase	Population/key findings	References
NCT00026312	Ongoing	Dinutuximab (anti-GD2 monoclonal antibody) + cytokines/retinoic acid	Phase III (randomized)	High-risk NB maintenance; dinutuximab significantly improved EFS/OS; established SoC	Yu et al., 2010; Desai et al., 2022
NCT03363373	Rrecruiting	Naxitamab (humanized anti-GD2 monoclonal antibody) + GM-CSF	Phase II	Relapsed/refractory NB; objective responses and durable remissions; FDA accelerated approval	Mora et al. (2025)
NCT03373097	Ongoing	GD2-CART01 (CAR-T cell therapy targeting GD2)	Phase I/II	High-risk NB/relapsed/refractory NB; feasible and safe, with durable CR in some patients	Yeku and Longo 2023; Locatelli et al., 2025
NCT00911560	Ongoing	GD2/GD3 vaccine (ganglioside-based vaccine) ± β-glucan	Phase II (randomized)	High-risk NB in remission; β-glucan enhanced anti-GD2 IgG1 responses, linked with improved PFS	Cheung et al. (2023)
NCT03107988	Completed	Lorlatinib (ALK tyrosine kinase inhibitor)	Phase I	Relapsed/refractory ALK-driven NB; meaningful responses, especially with chemo	Goldsmith et al. (2023)
NCT01175356	Ongoing	131I-MIBG (radiolabeled norepinephrine analogue) + Vorinostat (HDAC inhibitor)	Phase I	Relapsed/refractory NB; established feasibility and radiosensitizing potential	Weiss et al. (2021)
NCT02035137	Completed	131I-MIBG (radiolabeled norepinephrine analogue) combination strategies	Phase II	Relapsed/refractory NB; MIBG+vorinostat arm showed the highest ORR with manageable toxicity	DuBois et al. (2021)
NCT00567567	Completed	Tandem autologous transplant (high-dose chemotherapy + autologous stem cell rescue)	Phase III (randomized)	High-risk NB; tandem transplant improved EFS	Park et al. (2019)
NCT02395666	Completed	DFMO/Eflornithine (ornithine decarboxylase inhibitor; polyamine metabolism blockade) maintenance	Phase II (single arm with external control)	High-risk NB; PSM vs. ANBL0032 showed reduced relapse risk and improved EFS/OS	Oesterheld et al. (2024)
NCT01767194	Completed	Aurora-A inhibitor alisertib with irinotecan/temozolomide	Phase II (randomized), Children’s Oncology Group (COG)	Relapsed/refractory/progressive NB; irinotecan/temozolomide + dinutuximab/GM-CSF achieved ORR ∼40% with improved PFS/OS	Mody et al. (2020)

Abbreviations in Table 1: NB, neuroblastoma; EFS, Event-Free Survival; OS, Overall Survival; COG, Children’s Oncology Group; GM-CSF, Granulocyte-Macrophage Colony-Stimulating Factor; IL-2, Interleukin-2; FDA, food and drug administration; CR, Complete Response; PFS, Progression-Free Survival; ORR, Objective Response Rate; HDAC, histone deacetylase; MIBG, Metaiodobenzylguanidine; DFMO, Difluoromethylornithine (eflornithine); SoC, Standard of Care; PSM, propensity score matching; ALK, anaplastic lymphoma kinase.

## 3 Therapeutic approaches that indirectly target *MYCN*

**FIGURE 2:**
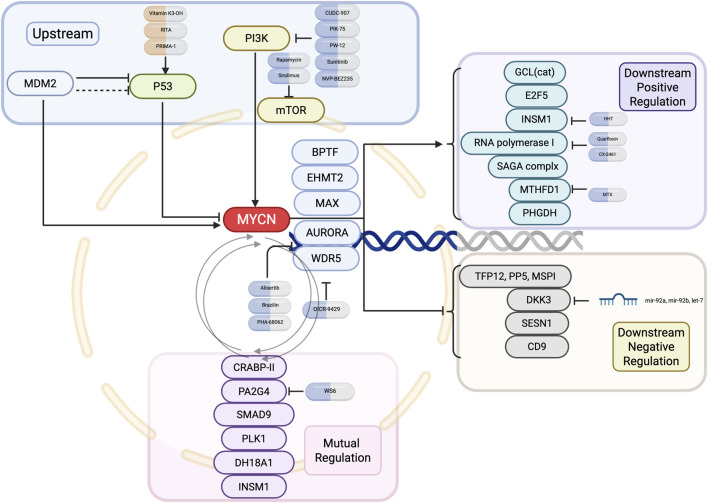
Upstream and downstream regulatory network of *MYCN* in neuroblastoma. Key upstream pathways, mutual regulators, and downstream positive and negative effectors of *MYCN* are shown, along with representative small-molecule inhibitors targeting each node.

While all MNA-NB are classified as high-risk neuroblastomas, there is still a lack of treatment options that directly target *MYCN* due to an insufficient understanding of the structure of the N-Myc protein (Bell et al., 2010; Wolpaw et al., 2021). Therefore, many studies have focused on the *MYCN* gene and its transcriptional regulatory products, as well as the interactions between *MYCN* and other genes and proteins, to identify new strategies for the treatment of MNA-NB. This review summarizes the current research progress of therapeutics that indirectly target *MYCN* for the treatment of neuroblastomato provide new ideas for the treatment of MNA-NB and other *MYCN*-amplified tumors. Figure 2 and Table 2 provide an overview of the *MYCN* regulatory network, including its upstream regulators, cooperating partners, downstream positive and negative targets, and mutual adjustment loops (Figure 2; Table 2).

**TABLE 2:** Upstream, downstream, and interacting genes of *MYCN*.

Category	Gene/target	Class	Mechanism of action	Drug/intervention	Experimental model	Outcome	References
Cooperating Effect	AURORA	Kinase	Forms complex with Aurora-A kinase to stabilize *MYCN*	HLB-0532259, PHA-68062	MNA-NB xenograft mouse	Significant tumor suppression	Tang et al., 2025, Boi et al., 2021
	ALK	Receptor tyrosine kinase	Dual inhibitor reduces *MYCN* expression	ESK44 (Phase I)	NB-1 cells	Inhibits tumor activity	Chugh et al., 2025
	MAX	Transcription factor	*MYCN* requires dimerization with MAX for oncogenic function	-	Primary NB tissue	Regulates oncogenic activity	Ferrucci et al. (2018)
	WDR5	Chromatin remodeler	Targets WBM/WIN sites to disrupt transcriptional complex	OICR-9429	*MNA-NB cells*	Synergistic growth inhibition	Han et al. (2023)
	BPTF	Chromatin remodeling factor	Interacts with N-Myc/CRC to regulate cell cycle	-	MNA-NB cells	Potential therapeutic target	Felipe et al. (2024)
	IGF2BP1	RNA-binding protein	Forms cooperative loop with *MYCN*	BTYNB+YM-155	MNA-NB cells	Synergistic inhibition	Hagemann et al. (2023)
	TCF4 (E2-2)	Transcription factor	Regulates FOXM1/E2F network through super-enhancers	-	Kelly cell line	Controls proliferation	Aljouda et al. (2025)
	SMARCE1	SWI/SNF complex	Regulated by *MYCN* via non-canonical E-box	-	MNA-NB cells	Inhibits proliferation	Hu et al. (2022)
	IRF2BP2	Transcriptional regulator	Activated by *MYCN*/MEIS2/HAND2 via SEs	-	MNA-NB cells	Maintains high proliferation	Chen et al. (2024a)
Upstream Regulators	P53	Tumor suppressor	Indirect *MYCN* suppression via p53 activation	PRIMA-1, RITA	*MNA-NB cells*	Delays progression	Mlakar et al. (2019)
	MDM2	E3 ubiquitin ligase	Regulates *MYCN* stability (p53-dependent/independent)	Nutlin-3, MI-63	TH-*MYCN* transgenic mice	Delays tumorigenesis	Chen et al. (2009)
	PI3K	Signaling kinase	Pathway inhibition reduces *MYCN* stability	CUDC-907, NVP-BEZ235	MNA-NB xenograft mouse	Induces degradation	Chanthery et al. (2012)
	AF1q	Oncoprotein	Regulates *MYCN* through Ras/ERK pathways	-	MNA-NB cells	Blocks cell cycle	Oskouian et al. (2024)
Promoter Regulation	CDK7/9/12	Cyclin-dependent kinases	Inhibits transcriptional elongation of *MYCN*	THZ1, YKL-5-124	MNA-NB xenograft mouse	Suppresses tumors	Gao et al. (2021)
	CRT	Chaperone protein	Binds *MYCN* promoter to inhibit transcription	Radiation	MNA-NB xenograft mouse	Promotes differentiation	Lee et al. (2019a)
	4-Oct	Transcription factor	Disruption induces caspase-2 apoptosis	-	MNA-NB cells	Reduces oncogenic transcripts	Nakatani et al. (2024)
	TFAP4	Transcription factor	Key effector of *MYCN* amplification	-	MNA-NB xenograft mouse	Promotes differentiation	Boboila et al. (2018)
	AHR	Nuclear receptor	Negatively regulates *MYCN* promoter	-	*MYCN* non-amplified cells and MNA-NB cells	Suppresses expression	Wu et al. (2014)
	BORIS	DNA-binding protein	Downregulation inhibits *MYCN* expression	-	MNA-NB xenograft mouse	Induces senescence	Rao et al. (2022)
	PP2A	Phosphatase	Dephosphorylates *MYCN*	ATUX-3364/8385	MNA-NB cells	Inhibits growth	Nazam et al. (2024)
Downstream (Activated)	GCL (cat)	Metabolic enzyme	*MYCN* activates via E-box binding (antioxidant pathway)	-	*MYCN* non-amplified cells and MNA-NB cells	Oxidative stress resistance	Veas-Perez de Tudela et al. (2010)
	E2F5	Transcription factor	Direct transcriptional activation by *MYCN*	-	MNA-NB cells	Promotes cell cycle	Liu et al. (2019)
	PHGDH	Metabolic enzyme	*MYCN*-driven serine metabolism	-	MNA-NB cells	Metabolic reprogramming	Arlt et al. (2021)
	MTHFD1	Metabolic enzyme	*MYCN*-activated folate metabolism	MTX+JQ1	*MYCN* non-amplified cells/MNA-NB cells/MNA-NB xenograft mouse	Therapy sensitization	Guan et al. (2024)
	INSM1	Transcription factor	Direct *MYCN* target	HHT	MNA-NB cells	Growth inhibition	Chen et al. (2023)
	SAGA complex	Epigenetic regulator	*MYCN*-dependent activity	KAT2A/B PROTAC	MNA-NB cells	Essential for *MYCN*	Malone et al. (2024)
Downstream (Repressed)	DKK1	Wnt inhibitor	Suppressed by *MYCN*	-	MNA-NB cells	Inhibits invasion	Koppen et al. (2007)
	DKK3	Wnt modulator	*MYCN*/miR-92-mediated suppression	Wnt agonists	MNA-NB cells	Promotes malignancy	Chen et al. (2024b)
	TFPI2	Protease inhibitor	Downregulated by *MYCN*	-	MNA-NB cells	Enhances invasion	Becker et al. (2008)
	SESN1	Stress-response protein	*MYCN*-regulated	-	MNA-NB cells	Inhibits migration	Hua et al. (2024)
	CD9	Surface glycoprotein	Transcriptional repression by *MYCN*/HDAC5	Vorinostat	*MYCN* non-amplified cells	Suppresses migration	Fabian et al. (2016)
Feedback Loops	INSM1	Transcription factor	Bidirectional: stabilizes *MYCN*, activated by *MYCN*	HHT	MNA-NB cells	Positive feedback	Chen et al., 2015,Chen et al., 2023
	PA2G4	Proliferation protein	Mutual stabilization with *MYCN*	WS6 analogs	MNA-NB cells	Induces apoptosis	Massudi et al. (2023)
	PLK1	Kinase	Phosphorylates SCF(Fbw7) to stabilize *MYCN*	-	MNA-NB cells	Enhances oncogenicity	Xiao et al. (2016)
	ALDH18A1	Metabolic enzyme	miR-29b/SP1 regulatory loop	YG1702	MNA-NB xenograft mouse	Tumor regression	Guo et al. (2020)
	SMAD9	Transcription factor	SE-mediated positive feedback with *MYCN*	-	MNA-NB cells	Promotes tumorigenesis	Tan et al. (2022)
	CRABP-II	Retinol-binding protein	Mutual activation with *MYCN*	-	MNA-NB cells	Positive feedback	Gupta et al. (2006)
	NCYM	Antisense gene	*MYCN*/OCT4 regulatory loop	ATRA	MNA-NB cells	Expression modulation	Kaneko et al. (2015)

### 3.1 Cooperating effect

*MYCN* cooperates with multiple oncogenic partners, and these synergistic interactions amplify tumorigenic signaling in NB. A series of studies have shown that *MYCN* has synergistic effects with genes such as *AURORA*, *ALK*, and *MDM2*.

#### 3.1.1 AURORA

Aurora-A kinase (AURKA) stabilizes N-Myc by preventing its proteasomal degradation, thereby sustaining oncogenic signaling in MNA-NB (Otto et al., 2009). Since N-Myc is undruggable, AURKA inhibition provides an indirect therapeutic strategy. The AURKA inhibitor alisertib (MLN8237) disrupts this interaction and promotes N-Myc degradation (Manfredi et al., 2011; Cervantes et al., 2012). Beyond single-agent activity, alisertib synergizes with BET inhibitors such as JQ1 to enhance N-Myc degradation and suppress tumor growth in preclinical models (Brockmann et al., 2013; Yi et al., 2021). Clinically, alisertib has been tested in combination regimens. In the Children’s Oncology Group phase I/II study (NCT01154816) (Mossé et al., 2019), alisertib with irinotecan and temozolomide was well tolerated and demonstrated clinical responses in relapsed or refractory NB in an international phase II trial (NCT01601535) (DuBois et al., 2018). These findings support AURKA inhibition as a promising indirect *MYCN*-targeting approach, particularly in combination with epigenetic or DNA-damaging agents currently under investigation.

#### 3.1.2 ALK

Activating *ALK* mutations, especially F1174L, frequently co-occur with *MYCN* amplification, synergistically accelerating neuroblastoma progression (Schönherr et al., 2012; Zhu et al., 2012; Hasan et al., 2013). Even without *ALK* mutations, activation of ALKAL2, a physiological ligand of ALK, enhances *MYCN*-driven tumorigenesis (Borenäs et al., 2021). ALK inhibitors provide a rational strategy. Clinically, NCT00939770 defined ALK inhibitor crizotinib dosing with partial responses (Foster et al., 2021), while NCT03107988 established the phase 2 dose of another ALK inhibitor lorlatinib (Goldsmith et al., 2023). An ongoing phase III trial (NCT03126916, ANBL1531) is testing lorlatinib in frontline high-risk NB (Weiss et al., 2021).

#### 3.1.3 MAX

N-Myc binds to E-box sequences in DNA by forming a heterodimer with MAX, thereby activating downstream oncogenic transcriptional programs, including genes involved in the cell cycle, metabolism, and proliferation (Wenzel and Schwab, 1995). The relative expression ratio of *MAX* to *MYCN* is a key factor in disease progression and clinical prognosis: a higher *MAX*/*MYCN* ratio can inhibit the oncogenic effects of *MYCN* and improve neuroblastoma prognosis (Lu et al., 2003). Therefore, MAX is not only a necessary binding partner for *MYCN* function, but may also play a balancing or antagonistic role in the *MYCN* regulatory network (Ferrucci et al., 2018).

#### 3.1.4 WDR5

As a key component of the N-Myc transcription complex, WDR5 participates in transcriptional regulation through its WBM site and mediates chromosome interactions through the WIN site. Han et al. screened 6 compounds targeting the WBM site, which were able to significantly inhibit the growth of the IMR32 and LAN5 cell lines with *MYCN* amplification and had a synergistic effect when combined with the WIN site inhibitor OICR-9429 (Han et al., 2023). Moreover, WDR5 promotes the recruitment of N-Myc to its conserved binding sites while recruiting DNA repair- and cell cycle-related genes to the N-Myc-WDR5 complex (Sun et al., 2015; Bumpous et al., 2023). WDR5 promotes the binding of N-Myc to the promoter region, while N-Myc recruits the histone methyltransferase G9a to the enhancer region to repress neuronal differentiation genes, including *GAP43* and *NRXN1*. These two cofactors thus cooperate to reinforce *MYCN*-driven transcriptional repression. Consistently, combined targeting of WDR5 and G9a has been shown to synergistically inhibit N-Myc activity and effectively block neuroblastoma growth (Liu et al., 2024).

#### 3.1.5 Other related cooperating genes

N-Myc acts synergistically with the core regulatory circuitry (CRC). Felipe et al. reported that the nucleosome remodeling factor BPTF interacts with N-Myc/CRC and colocalizes with the promoters of cell cycle genes, suggesting that BPTF is a potential therapeutic target for MNA-NB (Felipe et al., 2024). In MNA-NB, *IGF2BP1* (17q) and *MYCN* form a synergistic loop, promoting 2p/17q chromosome amplification and upregulating *BIRC5* expression. The BIRC5/survivin inhibitor YM155 can inhibit USP7 deubiquitinase activity, promote the degradation of N-Myc in MNA cells, and inhibit the growth of MNA-NB *in vivo* (Li et al., 2023). The combined use of BTYNB (an IGF2BP1 inhibitor), a BRD inhibitor, and YM-155 (a BIRC5 inhibitor) can synergistically inhibit the growth of MNA-NB cells, supporting its therapeutic potential (Hagemann et al., 2023). Studies by Aljouda et al. have shown that the class I basic helix-loop-helix transcription factor TCF4 (also known as E2-2), a component of the super enhancer (SE)-associated transcription factor network, regulates the *FOXM1*/*E2F-*mediated gene network and synergizes with the N-Myc protein to regulate the cell cycle progression and proliferation of neuroblastoma, playing a key role in the progression of NB (Aljouda et al., 2025). Chen et al. reported that *MYCN*, *MEIS2*, and *HAND2* activated *IRF2BP2* through SEs and that the AP-1 family was enriched at its binding sites, synergistically promoting *ALK* expression to maintain high proliferation of NB (Chen X. et al., 2024).

*SMARCE1*, as the coding gene of the SWI/SNF chromatin remodeling complex, regulates the expression of *MYCN* target genes such as *PLK1*, *ODC1*, and *E2F2*. In MNA-NB cells, *SMARCE1* knockdown inhibits cell proliferation and colony formation. Mechanistically, N-Myc directly regulates its transcriptional activity by binding to the nonclassical E-box in the promoter region of *SMARCE1* (Hu et al., 2022).

### 3.2 Upstream genes

Genes upstream of *MYCN* such as *TP53* and PI3K encoding genes, critically regulate its expression, thereby indirectly shaping tumor growth and therapeutic response in NB.

#### 3.2.1 TP53

The p53 activator PRIMA-1 rapidly induces oxidative stress and cell death in MNA-NB cells, which is caused by modulation of the methionine/cysteine/glutathione axis rather than induction of apoptosis (Mlakar et al., 2019). RITA, a p53 activator, activates p53 and triggers apoptosis, inhibiting the expression of *MYCN* (Burmakin et al., 2013). However, *MDM2* promotes neuroblastoma development by inhibiting p53. For example, in MDM2 haploinsufficient *MYCN* transgenic mice, tumor onset is delayed and survival is prolonged, and in human xenograft models, *MDM2* knockdown significantly inhibits tumor growth through a p53-dependent mechanism (Chen et al., 2009). In MNA-NB cells, *MDM2* can also regulate *MYCN* expression in a p53-independent manner, thereby affecting NB tumor growth (He et al., 2011). Gu et al. revealed that MDM2 binds to the ARE element of the *MYCN* mRNA 3′UTR through the C-terminal RING domain, enhancing mRNA stability and translation. In NB cells, *MDM2* overexpression can upregulate *MYCN* expression in a p53-independent manner (Gu et al., 2012). Agarwal et al. reported that N-Myc directly binds to the C-terminal domain of the p53 tetramer and that this effect is independent of the formation of the N-Myc/MAX heterodimer. The N-Myc-p53 complex targets genes involved in DNA repair and exacerbates carcinogenesis (Agarwal et al., 2018). Notably, vitamin K3-OH, a derivative of vitamin K3, can enhance the action of p53 in MNA-NB cells and inhibit the expression of *MYCN*, accompanied by a decrease in miRNA LIN28 (Yoda et al., 2019). Using the SHEP Tet21N *MYCN*-regulatable system, Gamble et al. reported that MNA-NB cells were more sensitive to apoptosis induced by the MDM2-p53 antagonists Nutlin-3 and MI-63 than those without high *MYCN* expression. These compounds may be particularly effective in treating high-risk *MYCN* amplification diseases (Gamble et al., 2012).

#### 3.2.2 PI3K encoding genes

High expression of PI3K and histone deacetylase (HDAC) is associated with poor OS in NB patients. CUDC-907, a dual inhibitor of PI3K and HDAC, can reduce *MYCN* levels in NB cells and increase H3K9Ac levels, promoting apoptosis (Chilamakuri and Agarwal, 2022). In *MYCN*-driven neuroblastoma and medulloblastoma mouse models, the PI3K inhibitors PIK-75 and PW-12 have been shown to induce tumor apoptosis by disrupting N-Myc stability (Cage et al., 2015). The receptor tyrosine kinase (RTK) inhibitor sunitinib decreases N-Myc protein levels by inhibiting PI3K/AKT signaling and GSK3 beta (Calero et al., 2014). The clinical PI3K inhibitor NVP-BEZ235 induces N-Myc degradation, inhibits angiogenesis, and prolongs survival in MNA-NB, including in humans and transgenic mouse models (Chanthery et al., 2012; Vaughan et al., 2016). In MNA-NB cells, PI3K inhibition downregulates N-Myc, while *MYCN* siRNA combined with rapamycin synergistically inhibits VEGF expression (Kang et al., 2008). The expression of the mTOR substrate-encoding gene eukaryotic translation initiation factor 4E-binding protein 1 (*EIF4EBP1*) is positively correlated with *MYCN* expression (Voeltzke et al., 2022). Moreover, the combination of the mTOR inhibitor temsirolimus and BET inhibitors (JQ1 or OTX-015) has a significant synergistic inhibitory effect on MNA-NB (Kling et al., 2021). AF1q is an oncoprotein, and *AF1q* gene knockdown can degrade N-Myc protein through the Ras/ERK and AKT/GSK3β pathways while activating p53 to block cell cycle progression. AF1q is a newly identified type of N-Myc regulatory factor (Oskouian et al., 2024).

#### 3.2.3 Regulation of the *MYCN* promoter

Delehouzé et al. demonstrated that the CDK inhibitors (R)-roscovitine and (S)-CR8 significantly reduce *MYCN* expression by blocking CDK7/9/12 and verified this effect in MNA-NB cells and a mouse transplant model (Delehouzé et al., 2014). THZ1, a CDK7 and SE inhibitor, inhibits *MYCN* gene transcription (Chipumuro et al., 2014). When combined with the tyrosine kinase inhibitors ponatinib and lapatinib, it reduces N-Myc protein expression by downregulating PNUTS (Tee et al., 2020). YKL-5-124 is a CDK7-specific inhibitor that is different from the multitarget inhibitor THZ1. In MNA-NB, YKL-5-124 induces cell cycle abnormalities and significantly inhibits tumor cell growth and the development of patient-derived xenografts when combined with the BRD4 inhibitor JQ1 (Gao et al., 2021).

Calreticulin (CRT) binds to the *MYCN* 5′ proximal promoter, which may overlap with the binding site of the transcription factor E2F1 and is an inhibitor of *MYCN*. CRT-mediated *MYCN* inhibition leads to increased differentiation of MNA-NB. Ionizing radiation increases CRT expression in NBs in a dose-dependent manner. The combined effect of these two factors can slow tumor growth in xenograft MNA-NB models (Lee et al., 2019). OCT4 is a transcription factor that regulates *MYCN* expression, and inhibiting its binding can induce caspase-2-dependent apoptosis in MNA-NB and reduce the high translation of *HNRNPA1/PTBP1* transcripts maintained by *MYCN*. Targeting OCT4-*MYCN* binding may be an effective therapeutic strategy for MNA-NB (Nakatani et al., 2024). Transcription factor activator protein 4 (TFAP4) is a key effector of *MYCN* amplification in neuroblastoma. Knockdown of *TFAP4* slows tumor growth and promotes tumor differentiation in a mouse xenograft model of MNA-NB (Boboila et al., 2018). Aryl hydrocarbon receptor (*AHR*) expression is negatively correlated with *MYCN* expression. *AHR* overexpression can inhibit *MYCN* promoter activity, whereas E2F1 overexpression can reverse this effect (Wu et al., 2014). As a nucleic acid-binding protein, the downregulation of Brother of Regulator of Imprinted Sites (*BORIS*) can inhibit the expression of B-cell-specific Moloney murine leukemia virus integration site (*Bmi1*), *Akt*, and *MYCN* and destroy telomere stability, leading to cell senescence. MNA-NB cells lacking *BORIS* lose their tumorigenic ability in xenograft models (Rao et al., 2022). Protein phosphatase 2A (PP2A) is a tumor suppressor protein. The PP2A activators ATUX-3364 and ATUX-8385 can lead to the dephosphorylation of the N-Myc protein at Serine 62 (S62) and reduce *MYCN* expression, resulting in slower growth of *MYCN*-amplified SK-N-BE (2) tumors (Nazam et al., 2024).

### 3.3 Downstream genes

Downstream effectors of *MYCN* govern proliferation, metabolism, and differentiation, making them key determinants of neuroblastoma progression. Many *MYCN*-related genes, such as *ACSL4*, *E2F5*, and *SKA3*, have been identified and described (Valentijn et al., 2012). The regulation of downstream genes by *MYCN* can be divided into two types: positive and negative.

#### 3.3.1 Positive regulation

*MYCN* regulates cell proliferation, oxidative stress, and metabolic reprogramming by directly binding to target gene promoters. Veas-Perez de Tudela et al. reported that *MYCN* regulates the expression of the gene encoding glutamate-cysteine ligase (GCL (cat)) by binding to the promoter E-box. Knockdown of *MYCN* increases the sensitivity of NB cells to oxidative damage, indicating that *MYCN* promotes tumor survival through an antioxidant mechanism (Veas-Perez de Tudela et al., 2010). N-Myc directly binds to the Myc E-Box within the *E2F5* gene promoter to induce its transcription. *E2F5* knockdown can inhibit cell cycle progression by regulating CDK2 and CDK6, thereby inhibiting the proliferation of MNA-NB cells (Liu et al., 2019). Chen et al. screened a plant alkaloid, homoharringtonine (HHT), which can effectively inhibit the *INSM1* promoter. Mechanistic studies have shown that HHT interferes with the binding of N-Myc to the *INSM1* promoter, inhibits PI3K/AKT-mediated N-Myc stability, and helps inhibit the growth of NB tumor cells (Chen et al., 2023).

In addition, *MYCN* maintains its oncogenic function by activating ribosome biogenesis and epigenetic regulation. *MYCN* upregulates ribosomal RNA and protein synthesis through transcription, whereas the RNA polymerase I inhibitors quarfloxin and CX-5461 can induce cell cycle arrest and apoptosis by inhibiting *MYCN* and activating p53. Among them, CX-5461 significantly inhibits tumor growth in the MNA-NB mouse model (Hald et al., 2019). Malone et al. identified through functional genomic screening that the Spt-Ada-Gcn5-acetyltransferase (SAGA) complex is a downstream target of *MYCN*. SAGA activity is positively correlated with *MYCN* expression and stability and can be used as a target for the proteolysis-targeting chimera (PROTAC) (Malone et al., 2024).

Targeting *MYCN* downstream metabolic enzymes or combining them with epigenetic drugs may increase their efficacy. *PHGDH* encodes the protein phosphoglycerate dehydrogenase and is associated with *MYCN* that is continuously expressed. *MYCN* binds to its two promoter regions. Inhibition of PHGDH can slow the proliferation of NB cells *in vitro*, but *in vivo* experiments have shown that it can also induce NB resistance to cisplatin (Arlt et al., 2021). Methylenetetrahydrofolate dehydrogenase 1 (MTHFD1) is positively correlated with *MYCN* expression, and ChIP‒qPCR and dual-luciferase reporter assays have revealed that *MYCN* directly activates *MTHFD1*. Knockdown of *MTHFD1* reduces the NADPH/NADP (+) and GSH/GSSG ratios, increases reactive oxygen spieces (ROS), and inhibits NB cell proliferation and migration. Knockdown of *MTHFD1* or the use of methotrexate (MTX) can enhance the antitumor effect of JQ1 both *in vitro* and *in vivo*, and combined treatment has potential for clinical translation (Guan et al., 2024).

#### 3.3.2 Negative regulation

In NB cell lines, Koppen et al. reported that the upregulation of *MYCN* can lead to the inhibition of Dickkopf-1 (DKK1) protein expression. The IMR32 cell line exhibited impaired proliferation when *DKK1* was overexpressed, which may be related to the regulation of *DKK1* by genes that inhibit cell proliferation and invasion, including *SYNPO2* (Koppen et al., 2007). J. Becker, S. et al. reported that in MNA-NB, the level of tissue factor pathway inhibitor 2 (TFP12, PP5, MSPI), which inhibits matrix metalloproteinases, was reduced, which may be related to the increased invasiveness of MNA-NB (Becker et al., 2008). Dickkopf 3 (*DKK3*) is a target site of basic helix-loop-helix (BHLH) transcription of the N-Myc. In neuroblastoma, the expression levels of *MYCN* and *DKK3* are negatively correlated. Validation via Wnt agonists and the p53 inhibitor PFT-alpha revealed that *MYCN* activates the Wnt/beta-catenin/Fra-1 pathway by inhibiting *DKK3* expression, ultimately inhibiting p53 activity and leading to the malignant progression of NB (Chen Y. et al., 2024). Haug et al. reported that *MYCN*-regulated miR-92a/b and let-7e can target the 3′ UTR of *DKK3* and confirmed their ability to inhibit *DKK3* transcription through luciferase reporter experiments; in NB cells, miR-92 also regulates the secretion of DKK3 (Haug et al., 2011). Hua et al. regulated the expression of *MYCN* and *SESN1* (a member of the Sestrin family and a target gene of p53) in NB cells by small interfering RNA (siRNA) or overexpression plasmids and confirmed that *SESN1* is regulated by *MYCN*. The overexpression of *SESN1* inhibited NB cell proliferation, migration, and invasion through the Toll-like receptor (TLR) signaling pathway (Hua et al., 2024). N-Myc colocalizes with HDAC5 at the promotor of the gene that encodes cell surface glycoprotein CD9, and represses its transcription. In SH-EP cells, *CD9* expression inhibits migration and invasion, whereas the HDAC inhibitor vorinostat induces *CD9* expression (Fabian et al., 2016).

### 3.4 Mutual adjustment or loop formation

Mutual adjustment refers to reciprocal regulation, where *MYCN* and a partner gene influence each other’s expression or stability. Loop formation, in contrast, represents a stable positive feedback circuit in which *MYCN* directly upregulates a factor that in turn reinforces *MYCN*, creating sustained oncogenic signaling.

Studies have shown that *MYCN* directly binds to the *CRABP-II* promoter to induce its transcription, while *CRABP-II* overexpression can increase N-Myc protein levels, forming a positive feedback regulatory loop (Gupta et al., 2006).

N-Myc can directly bind to the *proliferation-associated protein 2G4* (*PA2G4*) gene promoter and bind to the PA2G4 protein through a 14-amino acid sequence. Inhibiting N-Myc-PA2G4 binding effectively inhibits MNA-NB tumor growth and reduces the levels of both proteins (Koach et al., 2019). Moreover, PA2G4 can bind to N-Myc and increase its stability. WS6 and its low-toxicity analogs can bind to PA2G4, reduce N-Myc levels in MNA-NB cells, and induce apoptosis (Massudi et al., 2023).

*SMAD9* is an independent prognostic factor for high-risk neuroblastoma, and its expression is directly regulated by *MYCN* through SEs. Studies have confirmed that SMAD9 also binds to the *MYCN* promoter to form a positive feedback loop, and gene silencing experiments have shown that *SMAD9* knockout can significantly inhibit MNA-NB cell proliferation and tumor formation ability (Tan et al., 2022). Polo-like kinase-1 (PLK1) phosphorylates and ubiquitinates SCF (Fbw7), promoting its degradation and blocking the decomposition of N-Myc. Stable N-Myc can directly activate *PLK1* transcription, forming a positive feedback loop and enhancing the oncogenic activity of N-Myc (Xiao et al., 2016). Aldehyde dehydrogenase family 18 member A1 (ALDH18A1) can posttranscriptionally regulate *MYCN* expression through the miR-29b/SP1 autoregulatory loop, and *MYCN* can transactivate *ALDH18A1*. The ALDH18A1 inhibitor YG1702 causes tumor regression and prolongs survival in a patient-derived xenograft (PDX) model of NB (Guo et al., 2020). INSM1 inhibits N-myc phosphorylation (Thr58) and degradation through the PI3K/AKT/GSK3β pathway, while N-myc binds to the *INSM1* promoter and activates its expression (Chen et al., 2015). *MYCN* and its cis-antisense gene *NCYM* form a positive feedback loop with *OCT4*, and all-trans retinoic acid (RA) can reduce the expression of *MYCN*, *NCYM*, and *OCT4* in MNA-NB (Kaneko et al., 2015).

### 3.5 Immunotherapy

Immunotherapy has emerged as a critical strategy in MNA-NB, aiming to overcome immune evasion and improve clinical outcomes. Immunotherapy targeting GD2 is the current first-line treatment for MNA-NB. However, MNA-NB utilizes an immune escape mechanism involing epigenetic regulation and immune microenvironment remodeling. These findings provide a theoretical basis and translational ideas for optimizing the clinical treatment of MNA-NB.

#### 3.5.1 Targeting GD2

The inclusion of dinutuximab (a monoclonal antibody against GD2) in first-line therapy significantly improves survival, and its combination with chemotherapy is effective in recurrent disease. However, GD2 expression levels in TH-*MYCN* (a transgenic neuroblastoma model driven by tyrosine hydroxylase promoter–driven *MYCN* overexpression) NBs were previously considered too low for targeting, resulting in the inadequate use of this model for GD2-targeted therapy studies (McNerney et al., 2022). The HDAC inhibitor vorinostat induces the upregulation of GD2 on the surface of MNA-NB cells and has a synergistic effect on the inhibition of MNA-NB by an anti-GD2 mAb (Kroesen et al., 2016). Indoleamine-pyrrole 2,3-dioxygenase 1 (IDO1) inhibits immune cell activity through tryptophan metabolism, interferes with IFNγ production, and thus weakens the killing effect of GD2. Chimeric antigen receptor T cell (CAR-T)/NK (natural killer) cells on NBs. *MYCN* transcriptionally inhibits the *IDO1* promoter, suggesting that the IDO1 inhibitor BMS-986205 combined with GD2. CAR-T/NK cells have the potential to treat MNA-NB (Caforio et al., 2021). The multifunctional nanomedicine (ANM) constructed by Zhang et al. contains a GD2 aptamer, *MYCN* siRNA, and doxorubicin, which can target GD2 (+) IMR32 tumors and inhibit their growth *in vivo* (Zhang et al., 2021).

#### 3.5.2 Immune escape in MNA-NB

Elisa Brandetti et al. reported that in the *MYCN*-inducible Tet-21/N-cell line, downregulation of *MYCN* expression can lead to the upregulation of ligands such as MICA, ULBPs, and PVR, making NB more easily recognized by NK cells, thereby avoiding their own immune defense (Brandetti et al., 2017). *MYCN* mediates immune suppression by increasing chemokine-like factor (CKLF) secretion to recruit CCR4+CD4(+) T cells. Targeting the CKLF pathway can inhibit MNA-NB immune escape (Qin et al., 2024). Seier et al. reported that the H3K9 euchromatic histone-lysine methyltransferases (EHTM) 1 and EHMT2 inhibited the transcriptional response to IFN-γ in MNA-NB, whereas EHMT inhibitors increased IFN-γ-induced *CXCL9* and *CXCL10* expression and promoted T-cell infiltration. The combined use of EHMT and EZH2 inhibitors tazemetostat (EPZ-6438) or GSK126 promoted MNA-NB regression by increasing Th1 chemokine expression (Seier et al., 2021).

#### 3.5.3 Harnessing the immune system to kill MNA-NB

Himoudi et al. used the *MYCN*-derived HLA-A2-restricted peptide VILKKATEYV to stimulate T cells and obtained two CTL clones that could kill MNA-NB (Himoudi et al., 2008). Sussman et al. identified the target gene *calmodulin kinase-like vesicle-associated gene* (*CAMKV*) regulated by N-Myc in MNA-NB cells through ChIP-seq. This calcium-binding transmembrane protein is specifically expressed in the central nervous system and MNA-NB and is a potential nonblood–brain barrier-penetrating immunotherapy target (Sussman et al., 2020). Boboila et al. transplanted the neuroblastoma cell line 9464D derived from TH-*MYCN* transgenic mice into the kidneys of C57BL/6 mice to construct an MNA-NB metastasis model and confirmed that anti-PD1 antibodies combined with high-dose radiotherapy can inhibit tumor growth and enhance CD3(+) CD8(+) T-cell infiltration (Boboila et al., 2023). Grunewald et al. cocultured L1CAM-CAR-T cells with *MYCN*-induced neuroblastoma models and MNA-NB cell lines, performed RNA sequencing and public omics data analysis, and reported that high expression of *MYCN* inhibits L1CAM. The indirect *MYCN* inhibitor MLN8237 combined with L1CAM-CAR-T-cell therapy can enhance the killing effect on *MYCN*-overexpressing tumors (Grunewald et al., 2025).

CRISPR screening has been used to identify *H2AFY* as a PD-1 agent nivolumab resistance gene, and knocking out *H2AFY* reverses PD-1 blockade resistance in MNA-NB and promotes mesenchymal transformation (Nagarajan et al., 2024). The BET inhibitor JQ-1 promotes the infiltration of PD-1(+) CD8(+) T cells, CD4(+) T cells, and regulatory T (Treg) cells into TH-*MYCN* mouse neuroblastoma cells, and its combination with anti-PD-1 therapy can synergistically enhance the tumor inhibitory effect (Sauvage et al., 2022).

### 3.6 Epigenetic regulation

**FIGURE 3:**
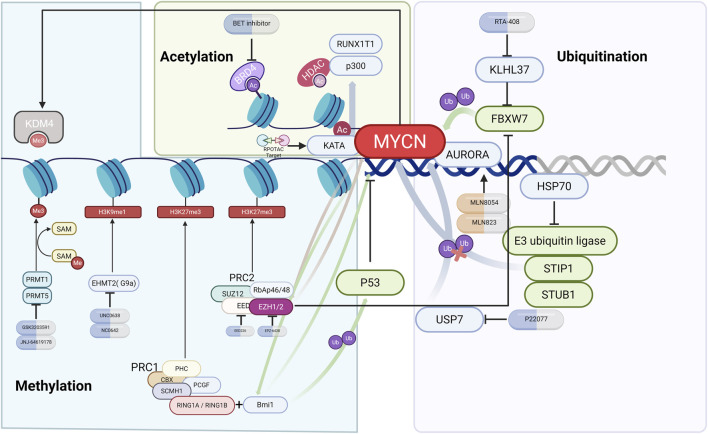
Epigenetic regulation of *MYCN* in NB. Schematic illustration of *MYCN* regulation by ubiquitination, acetylation, and methylation. Key modifiers (such as *AURKA*, USP7, *BRD4*, *EZH2*, *PRMT5*, and G9a) are shown together with representative inhibitors (e.g., alisertib, P22077, JQ1, tazemetostat, and UNC0638) and their effects on *MYCN* stability or transcriptional activity.

**FIGURE 4:**
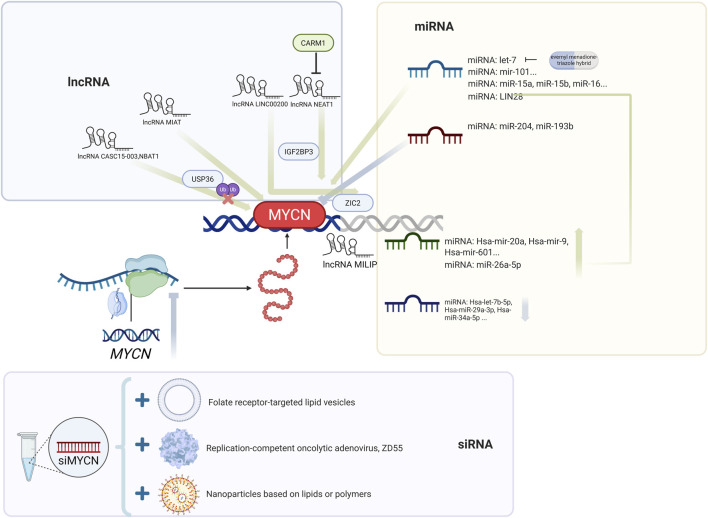
Post-transcriptional regulation of *MYCN* by noncoding RNAs. Schematic overview of *MYCN* regulation through lncRNAs (e.g., CASC15-003, NBAT1, MIAT, LINC00200, NEAT1, MILIP), miRNAs (e.g., let-7 family, miR-204, miR-193b), and siRNA-based strategies. Representative approaches such as miRNA mimics, si*MYCN*, antisense oligonucleotides (BGA002), and nanoparticle-mediated delivery are highlighted.

Epigenetic regulation controls the stability and activity of *MYCN*, shaping NB progression. This section highlights how ubiquitination, acetylation, methylation, and noncoding RNAs fine-tune *MYCN* expression. Figures 3, 4 and Table 3 summarize the major epigenetic mechanisms regulating *MYCN* stability, including ubiquitination, acetylation, methylation, and RNA-based regulation (Figures 3, 4; Table 3).

**TABLE 3:** Epigenetic modulation of *MYCN*.

Regulation type	Subcategory	Target/molecule	Mechanism of action	Intervention drug/method	Experimental model	Effect	References
Ubiquitination	-	Aurora kinase	Alters N-Myc/SCF(FbxW7) interaction	MLN8054/8237	MNA-NB cells	Inhibits N-Myc degradation	Brockmann et al. (2013)
	-	KLHL37	Blocks N-Myc/FBXW7 interaction	RTA-408	Patient-derived NB cells and MNA-NB xenograft mouse	Stabilizes N-Myc protein	Xiang et al. (2025)
	-	HSP70	Steric hindrance of STUB1-mediated ubiquitination	HSP70 allosteric inhibitors	MNA-NB cells	Promotes K416/K419 degradation	Xu et al. (2024)
	-	USP7 (HAUSP)	Mediates N-Myc deubiquitination	P22077	*MNA-NB xenograft mouse*	Suppresses tumor growth	Tavana et al. (2016)
Acetylation	-	BRD4	Recognizes acetylated histones to drive *MYCN* transcription	JQ1	MNA-NB cells	Downregulates *MYCN* expression	Puissant et al. (2013)
	-	HDAC3	Cooperates with *MYCN* to repress GRHL1	SAHA	MNA-NB cells	Induces apoptosis	Fabian et al. (2014)
	-	Runx1t1	Modulates chromatin accessibility via LSD1-CoREST3-HDAC	-	TH-*MYCN* transgenic mice	Suppresses NB initiation	Murray et al. (2024)
	-	p300	Maintains N-Myc stability via K199 acetylation	p300 inhibitors	MNA-NB cells	Reduces *MYCN* protein levels	Cheng et al. (2023)
		KAT2A	Acetylation enhances *MYCN* stability	KAT2A PROTAC degraders	MNA-NB cells and MNA-NB xenograft mouse	Decreases *MYCN* protein	Liu et al. (2025)
Methylation	-	PRMT1	Catalyzes asymmetric dimethylation	PRMT1 knockout	*MYCN non-amplified cells*	G2/M phase arrest	Eberhardt et al. (2016)
	-	PRMT5	Cooperates with E2F1 to regulate splicing factors	GSK3203591/JNJ-64619178	TH-*MYCN* transgenic mice	Induces apoptosis and extends survival	Bojko et al. (2024)
	-	EHMT2 (G9a)	Highly expressed in MNA-NB, suppresses differentiation	UNC0638/UNC0642	MNA-NB cells	Selectively induces apoptosis	Bellamy et al. (2020)
	-	KDM4A-C	*MYCN*-induced, maintains demethylated state	KDM4 inhibitors	MNA-NB cells	Promotes differentiation and apoptosis	Abu-Zaid et al. (2024)
	-	METTL3/METTL14/WTAP	Mediates m6A modification of HOX genes	METTL3 inhibitors	Neural crest models	Induces DNA damage and differentiation	Thombare et al. (2024)
	-	SNRPD3	*MYCN*/PRMT5 enhances its methylation	P-RMT5 inhibitors	MNA-NB cells	Inhibits BIRC5 aberrant splicing	Salib et al. (2024)
	-	KDM1A (LSD1)	Co-localizes with *MYCN* at CDKN1A/p21 promoter	TCP	MNA-NB cells	Restores tumor suppressor expression	Amente et al. (2015)
Non-coding RNA	lncRNA	CASC15-003	Regulates *MYCN* stability via USP36	USP36 inhibitors	MNA-NB xenograft mouse	Suppresses tumor growth	Juvvuna et al. (2021)
	lncRNA	MILIP	*MYCN*-activated, promotes DNA repair	MILIP inhibitors	MNA-NB cells	Enhances cisplatin efficacy	Wang et al. (2022b)
	lncRNA	NEAT1	Competes with miR-873-5p to upregulate *MYCN*	CARM1 inhibitors	MNA-NB cells	Inhibits proliferation and migration	Hu et al. (2025)
	miRNA	let-7 family	*MYCN*-repressed, targets *MYCN* mRNA	MX25-1	MNA-NB cells	Degrades *MYCN* mRNA	Liu et al. (2022b)
	miRNA	miR-204	Directly targets *MYCN* 3′UTR	REP-204	NCG mouse models	Inhibits tumor metastasis	Chiangjong et al. (2024)
	miRNA	miR-21	Downregulated by *MYCN* (no direct phenotype)	-	MNA-NB cells	No significant effect	Buechner et al. (2011a)
	miRNA	LIN28B	Activated by *MYCN* promoter	-	Human fetal sympathetic ganglia	Promotes invasion and migration	Tao et al. (2020)
	siRNA	si*MYCN*	Silences *MYCN* mRNA	Folate-nanoparticle delivery	Bone metastasis model	Suppresses metastasis	Zhu et al. (2013)
	siRNA	*MYCN*-A3	Alkylates *MYCN* transcript (pyrrole-imidazole polyamide)	Intravenous injection	MNA-NB xenograft mouse	Inhibits tumor progression	Yoda et al. (2019a)
	siRNA	Pt-191-PIP	Targets *MYCN* DNA sequence	IV (*in vitro* only)	Kelly cell line	Reduces cell viability	Obata et al. (2023)
Targeted Therapy	-	ZD55-sh*MYCN*	Oncolytic adenovirus + gene silencing	Intratumoral injection	MNA-NB cells	Induces apoptosis and oncolysis	Li et al. (2015)
	-	BGA002	Antisense PNA oligonucleotide targeting *MYCN*	Systemic administration	MNA-NB cells	Triggers mitochondrial damage	Montemurro et al. (2019)

#### 3.6.1 Ubiquitination

The protein kinase Aurora inhibits the stability of N-Myc by altering the way N-Myc interacts with SCF (FbxW7) and preventing its ubiquitination (Richards et al., 2016). The AURKA inhibitor MLN8054/8237 disrupts the AURKA/N-Myc interaction and promotes Fbxw7-mediated N-Myc degradation (Brockmann et al., 2013).

Kelch-like protein 37 (KLHL37) stabilizes N-Myc-promoting cancer by blocking the N-Myc/FbxW7 interaction, and its inhibitor RTA-408 can inhibit neuroblastoma both *in vivo* and *in vitro* (Xiang et al., 2025). Shock protein 70 (HSP70), a key chaperone protein of N-Myc, maintains the stability of N-Myc by blocking STUB1-mediated ubiquitination through steric hindrance; its allosteric inhibitor can promote the ubiquitination degradation of N-Myc at the K416 and K419 sites (Xu et al., 2024). Ubiquitin-specific protease 7 (USP7, HAUSP) can bind to N-Myc and induce N-Myc deubiquitination. The HAUSP small molecule inhibitor P22077 has been shown to significantly inhibit the growth of the MNA-NB xenograft mouse model (Tavana et al., 2016).

#### 3.6.2 Acetylation

BRD4, a member of the BET family, recognizes and binds to acetylated histones and drives *MYCN* transcription, whereas inhibitors such as JQ1 can displace BRD4 from the *MYCN* promoter region and downregulate *MYCN* expression (Puissant et al., 2013).

Cortés et al. reported that the HDAC inhibitor suberoylanilide hydroxamic acid (SAHA) induced apoptosis in all NB cell lines (regardless of *MYCN* status) and reduced *MYCN* expression, but changes in *MYCN* expression levels did not affect the efficacy of SAHA (Cortés et al., 2015). HDAC3 synergizes with *MYCN* to repress Grainyhead-like 1 (*GRHL1*) gene transcription, and *GRHL1* overexpression inhibits the proliferation and *in vivo* growth of MNA-NB, suggesting that *GRHL1* is a potential therapeutic target for HDAC inhibitors vorinostat (SAHA) or panobinostat (LBH589) (Fabian et al., 2014). Through large-scale mutagenesis screening, Murray et al. reported that knocking out *Runx1t1* can prevent the occurrence of neuroblastoma in transgenic mice. Although no direct interaction between *Runx1t1* and *MYCN* has been detected, the RUNX1T1 protein, a component of the LSD1-CoREST3-HDAC inhibitory complex, may inhibit the growth of NB with high *MYCN* expression by regulating chromatin accessibility through the inhibitory effect of the complex in the enhancer region (Murray et al., 2024). Cheng et al. identified 16 posttranslational modification sites and 114 interacting proteins through N-Myc coimmunoprecipitation and mass spectrometry analysis and reported that p300 maintains its stability by regulating the acetylation/ubiquitination of the N-Myc K199 site, suggesting that p300 is a potential target for N-Myc-targeted therapy (Cheng et al., 2023). Whole-genome sequencing of IMR32 cells revealed that N-Myc recruits the transcriptional coactivator KAT2A to bind to DNA, promoting the expression of genes related to ribosome biogenesis and RNA processing, whereas KAT2A increases the stability of N-Myc through acetylation. Moreover, PROTAC-mediated KAT2A degradation can reduce N-Myc levels (Liu et al., 2025).

#### 3.6.3 Methylation

Protein arginine methyltransferase 1 (PRMT1) catalyzes the formation of asymmetric dimethylarginine. Knockdown of the gene encoding PRMT1 can arrest the cell cycle at G (2)/M and increase the p53 protein level in the MNB-NB cell line SK-N-SH (Eberhardt et al., 2016; Lee et al., 2019). Bate-Eya et al. reported that protein arginine methyltransferase 5 (PRMT5) and the transcriptional regulator E2F1 cooperate with *MYCN* to regulate splicing factor genes, disrupt RNA splicing programs, and inhibit NB apoptosis. The PRMT5 inhibitor T1-44 or E2F1 inactivation promotes NB apoptosis (Park et al., 2015; Bate-Eya et al., 2025). The PRMT5 inhibitor GSK3203591 inhibits growth and induces apoptosis in neuroblastoma, accompanied by changes in the DNA damage response, epitranscriptome and metabolism, and reduces GLS expression. GSK3203591 also prolongs the survival of TH-*MYCN* mice and has potential for use in MNA-NB therapy (Bojko et al., 2024).

*Bmi1* is one of the core proteins of polycomb repressive complex 1 (PRC1) (Lin et al., 2015; Fitieh et al., 2021). Ochiai et al. confirmed the transcriptional regulation of Bmi1 by targeting the N-Myc binding site in the *Bmi1* promoter. The study revealed that *MYCN* was significantly positively correlated with *Bmi1* expression. Mechanistic studies have shown that *Bmi1* promotes the proliferation of neuroblastoma cells and inhibits their differentiation by binding to the promoter regions of the tumor suppressor genes *TSLC1* and *KIF1Bβ* (Ochiai et al., 2010). In NB, *Bmi1* also mediates the ubiquitination and degradation of p53 by binding to the polycomb complex protein Ring1A/Ring1B, leading to abnormal overexpression of the *MYCN* protein during embryonic development (Calao et al., 2013).

Embryonic ectoderm development (EED) is a component of polycomb repressive complex 2 (PRC2). EED knockout by shRNA, sgRNA, and the EED small-molecule inhibitor EED226 can significantly inhibit NB cell proliferation (Shaliman et al., 2022). Using CRISPR-Cas9 screening and ChIP analysis, Chen et al. reported that *MYCN* promotes *EZH2* expression by binding to the *EZH2* promoter, inhibiting neuronal differentiation in a PRC2-dependent manner, suggesting that EZH2 inhibitors tazemetostat (EPZ-6438) or GSK126 may be used to treat MNA-NB (Chen et al., 2018). Moreover, Wang et al. reported that EZH2 can compete with the SCF (Fbw7) ubiquitin ligase for N-Myc binding, counteracting Fbw7-mediated N-Myc polyubiquitination and proteasomal degradation. Knockdown of EZH2 can directly reduce the level of MN-Myc (Wang L. et al., 2022). Tsubota et al. reported that EZH2 and N-Myc are physically associated and that H3K27me3 expression is increased in the TH-*MYCN* mouse NB model. Moreover, the inhibitor EPZ-6438 can inhibit orthotopic tumor growth (Tsubota et al., 2017).

G9a is highly expressed in MNA-NB cells, and its knockout or inhibitors UNC0638 or UNC0642 selectively induce the apoptosis of MNA-NB cells. *MYCN* amplification enhances this effect, indicating that G9a is a potential therapeutic target for MNA-NB (Bellamy et al., 2020). *EZH1* knockout or UNC1999 inhibition have been shown to downregulate E2F target genes (*TYMS*, *POLA2*, *CCNA1*) by reducing H3K27me1 levels, and a reduction in *TYMS* (encoding thymidylate synthetase) significantly increased the sensitivity of MNA-NB to 5-FU (Shinno et al., 2022). Abu-Zaid et al. reported that *MYCN* can induce the expression of histone lysine demethylase 4 family members (*KDM4A-C*), and pharmacological inhibition of KDM4 can block *MYCN* expression and H3K9me3, inducing neuroblast differentiation and apoptosis (Abu-Zaid et al., 2024). METTL3/METTL14/WTAP mediate HOX gene m6A modification in neural crest cells, and HOX gene m6A modification in MNA-NB has been shown to lead to downregulation of HOX expression. METTL3 inhibition can induce DNA damage and differentiation in MNA-NB cells (Thombare et al., 2024). Salib et al. revealed that *MYCN* enhances the methylation of the core spliceosomal protein SNRPD3 by binding to SNRPD3 and PRMT5, thereby increasing its expression level and promoting the differential splicing of cell cycle factors such as BIRC5, forming a bidirectional regulatory loop. The PRMT5 inhibitor JNJ-64619178 can effectively inhibit the activity of MNA-NB cells (Salib et al., 2024). The chromatin-modifying enzyme lysine-specific demethylase 1 (KDM1A, LSD1) colocalizes with N-Myc in the promoter regions of *CDKN1A*/*p21* and *CLU*, and the use of the KDM1A inhibitor TCP can restore the expression of these two genes in MNA-NB cells (Amente et al., 2015).

#### 3.6.4 Long noncoding RNA (lncRNA)

Li et al. developed a new computational process, LncFusion. By analyzing the transcriptome data of three major childhood cancer cohorts, TARGET, Gabriella Miller Kids First, and St. Jude Cloud, they reported that MNA-NBs presented subtype-specific enrichment of lnc-fusion genes, suggesting that these genes may be involved in the pathogenesis of NB and providing a new target for MNA treatment (Li N. et al., 2025). Juvvuna et al. reported that the lncRNAs CASC15-003 and NBAT1 affect the stability of the N-Myc by regulating the deubiquitinase USP36. Downregulation of USP36 can significantly inhibit the growth of NB cells and transplanted tumors (Juvvuna et al., 2021). Downregulation of the lncRNA myocardial infarction-associated transcript (MIAT) reduces N-Myc expression and the *MYCN* target gene *ODC1* in MNA-NB and inhibits glycolysis, supporting its potential as a therapeutic target (Feriancikova et al., 2021). Chen et al. reported that the lncRNA LINC00200 is highly expressed in MNA-NB tissues and that its overexpression promotes the expression of the *MYCN* target gene *ZIC2* by binding to IGF2BP3, thereby increasing the proliferation, invasion, and migration ability of NB cells (Chen et al., 2022). Hu et al. confirmed through a luciferase reporter gene that the lncRNA NEAT1 acts as a competitive sponge for miR-873-5p, increasing *MYCN* and *GalNAcT-I* expression, whereas the inhibition of CARM1 can reduce NEAT1 activity (Hu et al., 2025). N-Myc promotes DNA repair by transcriptionally activating the lncRNA MILIP. MILIP knockout leads to the accumulation of DNA double-strand breaks (DSBs), induces apoptosis, and inhibits the proliferation of MNA-NB cells. Targeted inhibition of MILIP enhances the *in vivo* growth inhibitory effect of cisplatin on neuroblastoma (Wang P. L. et al., 2022).

#### 3.6.5 microRNA (miRNA)

Mestdagh et al. investigated the miRNA signature of *MYCN* using primary NB tumor data and reported that *MYCN* causes extensive miRNA repression by binding to the promoter region of targeted miRNAs and that these miRNA pathways are conserved in multiple tumors (Mestdagh et al., 2010). Through genome-wide miRNA target screening combined with patient data, 12 miRNAs that target and inhibit *MYCN* were identified. In the TH-*MYCN* transgenic mouse model, 9 of these miRNAs, including hsa-let-7a-5p, hsa-let-7b-5p, and hsa-miR-29a-3p, were expressed at low levels in tumor tissues, suggesting that *MYCN* maintains its high expression by inhibiting these miRNAs (Beckers et al., 2015a).

J. Buechner et al. predicted the miRNA targets of the *MYCN* 3′UTR through bioinformatics and verified them in neuroblastoma cells with *MYCN* amplification. They reported that the overexpression of let-7 and miR-101 significantly inhibited tumor cell proliferation and colony formation ability (Buechner et al., 2011). Liu et al. reported that the small molecule MX25-1 promoted the degradation of *MYCN* mRNA by binding to its 3′UTR, and this process was dependent on let-7 regulation (Liu F. et al., 2022). Szewczyk et al. constructed a tTR-KRAB repressor protein-regulated GFP reporter system and reported that kenpaullone and BIO inhibited N-Myc expression by enhancing let-7 miRNA expression (Szewczyk et al., 2024). Mendonza et al. constructed six hybrid compounds by simulating the classic structure of HDAC inhibitors, connecting evernyl groups with menadione via triazole linkers. Among them, Compound 6b significantly inhibited the proliferation of neuroblastoma by a mechanism involving the induction of the expression of microRNAs, including let-7, and thus downregulating N-Myc. In addition, Compound 6b enhanced the differentiation effect of RA combined therapy (Mendonza et al., 2023).

Chava et al. predicted and verified that miR-15a/15b/16 targets *MYCN* mRNA through databases such as TargetScan and that its overexpression significantly inhibits neuroblastoma cell proliferation, migration, invasion, and tumorigenesis in NCG mice (Chava et al., 2020).

J. Buechner, J. R et al. reported that knocking down *MYCN* in SK-N-BE cells led to the downregulation of 12 miRNAs (including mir-21), but artificial regulation of mir-21 levels (overexpression or inhibition) did not significantly affect the differentiation or proliferation of neuroblastoma cells with high *MYCN* levels (Buechner et al., 2011). LIN28 can increase the invasion and migration of MNA-NBs (Tao et al., 2020). In *MYCN*-amplified NB, *MYCN* can also increase the expression of the oncogene LIN28B by directly acting on the promoter and indirectly regulating miR-26a-5p (Beckers et al., 2015b). The *MYCN*-induced miRNA signature of NB cells revealed that miR-18a and miR-19a promoted NB development by downregulating estrogen receptor α (ESR1) expression and inhibiting the differentiation of human fetal sympathetic ganglia (Lovén et al., 2010). Interfering with miR-18a or overexpressing ERα can induce nerve growth factor (NGF) signaling and promote the differentiation of MNA-NB cells into neurons (Dzieran et al., 2018). miR-204 can directly bind to *MYCN* mRNA and inhibit *MYCN* expression (Ooi et al., 2018). Chiangjong et al. synthesized erythrocyte extracellular vesicles (REP-204) loaded with miR-204. SWATH proteomics revealed that REP-204 inhibited mRNA splicing and protein synthesis through the spliceosome, thereby inhibiting the growth and migration of neuroblastoma (Chiangjong et al., 2024). Introduction of miR-193b into different NB cell lines significantly inhibited cell growth by reducing the expression of *MYCN*, *Cyclin D1*, and *MCL-1*. Therefore, miR-193b may be a new candidate drug for MNA-NB (Roth et al., 2018).

#### 3.6.6 Small interfering RNA (siRNA)

In MNA-NB cell lines, silencing *MYCN* with siRNA molecules can promote cell differentiation, and this effect is better than that of RA. si*MYCN* may therefore become a new treatment for RA-resistant MNA-NB (Maeshima et al., 2020). The efficiency of folate receptor-targeted liposome delivery of *MYCN* siRNA to LA-N-5 cells reached 92%, which effectively silenced *MYCN* expression (Feng et al., 2010). The *MYCN*-specific antigene PNA oligonucleotide BGA002 can reduce the level of N-Myc protein in MNA-NB *in vitro* and *in vivo*, causing severe mitochondrial damage, thereby increasing ROS and inducing NB apoptosis (Montemurro et al., 2019). Folate-nanoliposome-entrapped *MYCN* siRNA can cause decreased *MYCN* expression and apoptosis of metastatic NBs in the bone marrow and bone metastasis xenograft mouse MNA-NB model (Zhu et al., 2013). Tagalakis et al. developed lipid-based and polymer-based nanoparticles to deliver siRNA targeting *MYCN* intravenously, effectively inhibiting the growth of neuroblastoma xenografts and prolonging the survival of mice (Tagalakis et al., 2021). The ZD55-sh*MYCN* oncolytic adenovirus blocks the cell cycle and induces apoptosis in MNA-NB cells by inhibiting *MYCN* expression and directly promoting cancer cell lysis (Li et al., 2013; Li et al., 2015).

Except for the interfering small RNA, the *MYCN*-targeting pyrrole-imidazole polyamide (PIP) *MYCN*-A3 can directly bind to and alkylate *MYCN* transcripts, downregulating *MYCN* expression and inhibiting tumor progression in the MNA-NB mouse xenograft suppressor model (Yoda et al., 2019). Obata et al. developed a new platinum-191-labeled compound based on pyrrole-imidazole polyamide (PIP), which can specifically bind to DNA containing the *MYCN* gene target sequence and significantly inhibit the viability of the neuroblastoma cell line Kelly of with high *MYCN* expression. However, animal experiments have shown that the uptake rate of this compound in tumor tissue after intravenous injection is low, limiting its clinical application value (Obata et al., 2023).

### 3.7 Metabolism-related targets

*MYCN* amplification drives profound metabolic reprogramming in NB, creating vulnerabilities across nucleotide, lipid, amino acid, glucose, and ferroptosis pathways. Through combined metabolome‒transcriptome analysis, Du et al. reported that, compared with non-MNA-NBs, MNA- NBs presented three abnormal pathways related to glycerolipid metabolism, purine metabolism, and lysine degradation, which can serve as potential therapeutic targets (Du et al., 2024). The following section summarizes *MYCN*-related metabolic targets (Table 4).

**TABLE 4:** *MYCN*-associated metabolic pathways.

Metabolic pathway	Target/molecule	Mechanism	Intervention	Experimental model	Effects	Reference
Nucleotide Metabolism	GART	Aberrant purine metabolism in *MYCN*-amplified NB (MNA-NB)	Lometrexol (inhibitor)	MNA-NB cells	Induces differentiation and inhibits tumor progression	Jiang et al. (2025)
	DHODH	*MYCN* upregulates DHODH to promote pyrimidine synthesis	DHODH knock down or Brequinar, GSK983 (inhibitors)	Xenograft/transgenic mice	Delays tumor growth; combined with dipyridamole overcomes resistance	Yu et al., 2021b, Olsen et al., 2022
Lipid Metabolism	ELOVL2	*MYCN* recruits PRC1 to catalyze H2AK119ub and suppress ELOVL2	-	Multi-omics analysis	Reveals *MYCN*’s key role in lipid metabolism regulation	Ding et al. (2019)
	SLC27A2 (FATP2)	*MYCN* upregulates FATP2 to enhance fatty acid uptake	SLC27A2 inhibition	Mouse models	Blocks tumor growth	Tao et al. (2022)
	GLDC	*MYCN* transcriptionally regulates GLDC for glycine cleavage	GLDC knockdown	MNA-NB cells	Inhibits proliferation, induces G1 arrest, disrupts purine/lipid metabolism	Alptekin et al. (2019)
Amino Acid Metabolism	PHGDH	Rate-limiting enzyme for serine synthesis; higher demand in MNA-NB	PHGDH knockdown or NCT-503 (inhibitor)	MNA-NB cells and PDX models	Inhibits proliferation but may antagonize cisplatin	Arlt et al. (2021)
	*MYCN*-ATF4 feedback loop	*MYCN* and ATF4 form positive feedback to stabilize *MYCN* protein	-	MNA-NB cells	Counteracts FBXW7-mediated ubiquitination	Xia et al. (2019)
	Polyamine metabolism	*MYCN* elevates polyamines to suppress Let-7 miRNA	DFMO (inhibitor)	MNA-NB cell and TH-*MYCN* mice	Restores LIN28/Let-7 axis, inhibits tumor growth, induces G1 arrest	Powers et al., 2016, Koomoa et al., 2013
	ATP13A3	Mediates polyamine uptake and DFMO resistance	AMXT 1501 (combined with DFMO)	MNA-NB cells	Enhances polyamine depletion	Azfar et al. (2025)
	Arg/Pro restriction diet	Reduces ornithine to potentiate DFMO effects	Arg/Pro-free diet	TH-*MYCN* mice	Significantly extends survival	Cherkaoui et al. (2025)
Glucose Metabolism and Respiration	Glycolytic reprogramming	*MYCN* amplification induces glycolytic dependence	Overexpression of *MYCN* and 2-Deoxyglucose	NB cell lines	Suppresses glycolysis	Tjaden et al. (2020)
	Mitochondrial remodeling	*MYCN* alters mitochondrial network for energy adaptation	-	MNA-NB cells	Adapts to metabolic demands	Casinelli et al. (2016)
	MCT1-Complex I co-inhibition	MNA-NB sensitivity to MCT1 (AZD3965) and Complex I (phenformin) inhibition	AZD3965 + phenformin	MNA-NB cells	Disrupts glycolysis and ATP production	Dalton et al. (2021)
	Mitoribosomal inhibition	Doxycycline activates ISR to degrade N-Myc	Doxycycline	MNA-NB cells	Sustained proliferation inhibition without resistance	Borankova et al. (2023)
	Mitochondrial uncoupler	Niclosamide ethanolamine (NEN) disrupts mitochondrial metabolism	NEN (dietary supplement)	Orthotopic NB mice	Downregulates N-Myc and induces differentiation	Byrne and Bell 2023, Jiang et al., 2023
	ClpXP protease inhibition	ONC201 reduces mitochondrial membrane potential	ONC201	MNA-NB cells	Promotes neurite outgrowth and differentiation	Wu et al. (2023)
Ferroptosis	SELENOP-LRP8 axis	LRP8 protects MNA-NB by promoting selenocysteine synthesis	-	CRISPR screen	Confers ferroptosis resistance	Alborzinia et al. (2023)
	SCLY-SEPHS2-PRDX6 network	PRDX6 interacts with SEPHS2 for selenium delivery	-	MNA-NB subtype analysis	Associated with specific MNA-NB subtypes	Chen et al. (2024c)
	Glutathione metabolism	MNA-NB relies on cysteine metabolism to maintain GSH levels	BSO (inhibitor)	TH-*MYCN* mice	Delays tumor growth	Carter et al., 2016, Floros et al., 2021
	TfR1 (CD71) regulation	*MYCN* upregulates TfR1 to accumulate iron and increase ferroptosis sensitivity	Gambogic acid (GA)	MNA-NB cells/mice	Induces apoptosis via NK-IRE1-mTORC1 pathway	Lu et al., 2021, Bishayee et al., 2019
	Prognostic model	Ferroptosis-related target-based prediction model for MNA-NB	-	Clinical data analysis	Potential therapeutic target screening	Tan et al. (2025)

#### 3.7.1 Nucleotide metabolism

Using mass spectrometry metabolomics and public RNA sequencing data, Jiang et al. reported that MNA-NBs purine metabolism enzyme-encoding genes were abnormal compared with non-MNA-NBs. Lometrexol, an inhibitor of the purine biosynthesis enzyme phosphoribosylglycinamide formyltransferase (GART), can induce differentiation in MNA-NB and inhibit tumor progression (Jiang et al., 2025).

The mitochondrial membrane protein dihydroorotate dehydrogenase (DHODH) is a key enzyme in pyrimidine synthesis, and *MYCN* promotes the production of pyrimidine nucleotides by upregulating the expression of *DHODH*. Knocking down *DHODH* or using inhibitors such as brequinar and GSK983 can delay the growth of MNA-NB tumors. Blocking uridine transport with dipyridamole can overcome the serum uridine-dependent resistance of DHODH inhibitors (Yu Y. et al., 2021). DHODH is regulated by *MYCN* in MNA-NB, and its high expression is significantly associated with poor patient prognosis. Brequinar effectively inhibits the growth of neuroblastoma in both transplanted tumors and transgenic mouse models, supporting its therapeutic potential (Olsen et al., 2022).

#### 3.7.2 Lipid metabolism

Through multiomics analysis, Ding et al. reported that *MYCN* inhibits *ELOVL fatty acid elongase 2 gene* (*ELOVL2*) expression by recruiting PRC1 to catalyze H2AK119ub, thereby reducing docosahexaenoic acid synthesis, revealing the key role of *MYCN* in the regulation of lipid metabolism in neuroblastoma (Ding et al., 2019). Through multidimensional metabolic analysis, it was found that MNA-NB is highly dependent on fatty acid metabolism. *MYCN* promotes fatty acid uptake by upregulating fatty acid transport protein 2 (FATP2), which is encoded by *SLC27A2*. Inhibition of *SLC27A2* can block tumor growth in mice, supporting the therapeutic potential of targeting fatty acid metabolism (Tao et al., 2022). Glycine decarboxylase (GLDC) is a key enzyme for glycine decomposition and is directly regulated by *MYCN* at the transcriptional level; its knockdown can inhibit MNA-NB cell proliferation, induce G1 phase arrest, disrupt purine metabolism, and reduce cholesterol and fatty acid levels, indicating that GLDC can be used as a therapeutic target for MNA-NB (Alptekin et al., 2019).

#### 3.7.3 Amino acid metabolism

Phosphoglycerate dehydrogenase (PHGDH) is the rate-limiting enzyme for serine synthesis. Isotope metabolomics have indicated that MNA-NB cells have a greater demand for serine synthesis than non-MNA-NB cells do. Serine/glycine starvation impairs only the nucleotide pool and proliferation of diploid *MYCN* cells and is ineffective against *MYCN*-amplified cells. *PHGDH* knockdown or the inhibitor NCT-503 inhibits MNA-NB cell proliferation. However, in the MNA-NB mouse PDX model, PHGDH inhibition antagonized the efficacy of cisplatin, suggesting that its therapeutic value is limited (Arlt et al., 2021). Moreover, the transcriptional activation of the serine metabolism pathway in MNA-NB cells requires *MYCN* and *ATF4* to form a positive feedback loop, antagonizing Fbxw7-mediated N-Myc ubiquitination to stabilize the N-Myc (Xia et al., 2019).

Spermidine and spermine are the major polyamines whose levels are elevated in MNA-NBs, leading to the downregulation of the tumor suppressor Let-7 miRNA via the upregulation of LIN28 (Powers et al., 2016). Difluoromethylornithine (DFMO), an ornithine decarboxylase inhibitor, has been shown to reduce polyamine levels, restore the balance of the LIN28/Let-7 axis, and inhibit tumor growth (Jiang and Yu, 2024). DFMO also induces p27(Kip1)/Rb-dependent G1 arrest in MNA-NB cells through accumulation of the p27(Kip1) protein (Koomoa et al., 2013). Azfar et al. reported that ATPase 13A3 (ATP13A3) promoted polyamine uptake under the action of the polyamine inhibitor DFMO and that AMXT 1501 inhibited this effect. The combination of these two drugs may become a new treatment for NB (Azfar et al., 2025). Cherkaoui et al. reported that a diet without arginine/proline reduced the content of the polyamine precursor ornithine and enhanced the depletion of tumor polyamines by DFMO, leading to ribosome stalling at adenylate-terminal codons. Dietary restriction of upstream amino acid substrates has been shown to significantly improve survival in the TH-*MYCN* mouse model (Cherkaoui et al., 2025).

#### 3.7.4 Glucose metabolism and cellular respiration

Exogenous overexpression of *MYCN* in NB cell lines can cause metabolic reprogramming of NB cells and sensitivity to the glycolysis inhibitor 2-deoxyglucose (Tjaden et al., 2020).

*MYCN* amplification in neuroblastoma induces structural changes in the mitochondrial network to adapt to altered energy demands (Casinelli et al., 2016). The antiprotozoal drug nifurtimox can increase oxidative stress in NB cell lines, reduce the activity of lactate dehydrogenase in anaerobic respiration, and reduce the expression of *MYCN* (Cabanillas Stanchi et al., 2015). Monocarboxylate transporters (MCTs) consist of four members (MCTs 1–4). Through metabolic targeted screening, Dalton et al. reported that MNA-NB cells are highly sensitive to the combined effects of the lactate transporter MCT1 inhibitor AZD3965 and complex I of the mitochondrial inhibitor phenformin, which can induce a sharp interruption in glycolysis and ATP production (Dalton et al., 2021).

The mitoribosomal inhibitor doxycycline can activate the mitochondrial stress response (MSR), inhibit protein synthesis, and promote N-Myc protein degradation. In MNA-NB cells, doxycycline can continuously reduce N-Myc levels and inhibit cell proliferation, and repeated administration does not induce drug resistance, indicating that it has a sustained therapeutic effect on MNA-NB (Borankova et al., 2023). Similarly, the mitochondrial uncoupler niclosamide ethanolamine (NEN) inhibits the growth of MNA-NB. NEN induces differentiation by disrupting mitochondrial metabolism and downregulating N-Myc, but its pharmacokinetic properties are poor and require further optimization (Byrne and Bell, 2023). The inclusion of NEN in the diet reduced the tumor growth rate and the expression of N-Myc and β-catenin in tumors in an orthotopic neuroblastoma mouse model (Jiang et al., 2023). ONC201, an inhibitor of the mitochondrial ClpXP proteases ClpP and ClpX, reduces the mitochondrial membrane potential of MNA-NB, and knocking down the ClpP and ClpX genes promotes neurite outgrowth. ONC201 treatment significantly reduces the expression of *NYCN*, promotes the differentiation of MNA-NB, and is a potential drug for the treatment of MNA-NB (Wu et al., 2023).

#### 3.7.5 Ferroptosis

Ferroptosis is an iron dependent, lipid peroxidation-driven form of programmed cell death that is closely related to tumorigenesis, development, and treatment resistance (Liu et al., 2020; Su et al., 2020).

Cheng et al. combined data from the ArrayExpress database with the Gene Expression Omnibus and FerroptosisDB websites and reported that *MYCN* is a key gene in NB regulation (Cheng et al., 2024). The predictive model developed by Tan et al.can predict the prognosis of MNA-NB using ferroptosis-related intervention targets and is expected to become a new therapeutic target for MNA-NB (Tan et al., 2025). Using CRISPR screening, Alborzinia et al. reported that the selenoprotein P (SELENOP) receptor LRP8 protects MNA-NB cells by promoting selenocysteine synthesis and helping to produce the anti-ferroptotic GPX4 (Alborzinia et al., 2023). Selenocysteine lyase (SCLY) uses SELENOP to synthesize selenide as a substrate for selenophosphate synthase 2 (SEPHS2). The (H(2)SePO(3) (−)) synthesized by SEPHS2 is used as a substrate for the synthesis of Sec-tRNA. Chen et al. reported that peroxidase 6 (PRDX6) can interact with SEPHS2 independently of SCLY, providing a selenium delivery system. Moreover, increased RDX6 expression is significantly associated with a subtype of MNA-NB (Chen Z. et al., 2024).

Neuroblastomas in TH-*MYCN* mice show stronger glutathione anabolism than those in wild-type mice, and preventive treatment with the glutathione biosynthesis inhibitor buthionine sulfoximine (BSO) delays tumor growth (Carter et al., 2016). Alborzinia et al. reported that MNA-NB depends on cysteine metabolism and that its deficiency can induce ferroptosis. MNA-NB maintains glutathione (GSH) levels by regulating cysteine metabolism and transsulfurization. In a mouse model, inhibiting cysteine uptake, transsulfurization, and glutathione peroxidase 4 (GPX4) activity can inhibit NB growth (Floros et al., 2021). *MYCN* can also increase the expression of transferrin receptor 1 (TfR1, CD71), leading to the accumulation of intracellular divalent iron ions and increasing the sensitivity of MNA-NB to ferroptosis induction (Lu et al., 2021; Alborzinia et al., 2022). Gambogic acid (GA), the natural ligand of TfR1, can also induce MNA-NB apoptosis through the NK-IRE1-mTORC1 pathway (Bishayee et al., 2019).

### 3.8 Adjunctive therapy

Adjunctive approaches aim to enhance the efficacy of existing therapies for MNA-NB, particularly by overcoming resistance to retinoic acid (RA) and other conventional agents. For existing MNA-NB or existing broad-spectrum tumor chemotherapy drugs, several studies have aimed to increase the effectiveness of these agents.

#### 3.8.1 Retinoic acid

Retinoic acid (RA) can bind to NB cells, reduce the level of N-Myc in NBs, induce NB differentiation, and inhibit proliferation (Haussler et al., 1983; Thiele et al., 1985). However, NB can be resistant to RA (Bates et al., 1989; Matsumoto et al., 1994).

Vasoactive intestinal peptide (VIP) significantly reduces *MYCN* expression in neuroblastoma cells (SH-SY5Y/IMR-32) and exhibits a synergistic inhibitory effect when combined with RA (Chevrier et al., 2008). Inhibition of transforming growth factor β (TGF-β) signaling is one of the mechanisms of MNA-NB resistance to retinoids. The combination of RA and the TGF-β activator kartogenin can inactivate RA-resistant MNA-NB cells (Duffy et al., 2017). Otsuka et al. reported that the tenascin-C-derived peptide TNIIIA2 combined with RA reduced neuroblastoma N-Myc protein levels, promoted differentiation, and inhibited tumor growth in a mouse model (Otsuka et al., 2019). BGA002 is a *MYCN*-specific antisense oligonucleotide that can synergistically inhibit *MYCN* when combined with RA. This combination therapy promotes autophagy by inhibiting the mTOR pathway and effectively inhibits tumor angiogenesis in the MNA-NB mouse model, significantly prolonging survival (Lampis et al., 2022). Broso et al. reported that isorhamnetin (ISR) synergistically inhibited MNA-NB cell viability by upregulating ADRA1B expression and retinoid isotretinoin. The α1 antagonist doxazosin also enhanced the antitumor effect of RA in a transplanted tumor model, indicating that inhibition of α-adrenaline receptors can enhance the growth inhibition and differentiation-promoting effects of RA (Broso et al., 2023). The aryl hydrocarbon receptor (AhR) inhibitor clofazimine can synergize with RA both *in vivo* and *in vitro* to induce MNA-NB differentiation (Chaudhry et al., 2023). RA induces the expression of the RA-metabolizing enzymes CYP26A1 and CYP26B1 in both *MYCN*-amplified Kelly and *MYCN*-nonamplified SH-SY5Y cells. RA combined with the selective CYP26 inhibitor talarozole or the RA-degrading enzyme CYP3A inhibitor ketoconazole can significantly reduce cell viability. The combination of RA with metabolic or HGF signaling pathway inhibitors may prevent the development of RA-resistant neuroblastoma (Issa et al., 2024).

## 4 Conclusion and prospects

*MYCN* plays a very important role in NB as a proto-oncogene. For clinicians and basic researchers, the undruggable nature of *MYCN* is a difficult problem to address. Fortunately, some research progress has been made on molecules directly related to *MYCN*.

Pandher et al. reported that the *MYCN* selective inhibitor M606 reduced N-Myc levels by binding to its promoter, upregulated hypoxia-inducible factor 1 alpha (HIF1A), and delayed the progression of NB in TH-*MYCN* mice (Pandher et al., 2025). The small molecule inhibitor 10058-F4 and its analogs developed by Müller et al. can bind to the C-Myc bHLHZip dimerization domain and inhibit the C-Myc/MAX interaction, inducing the apoptosis of MNA-NB cells (Müller et al., 2014). MYCMI-7, developed by Castel et al., can specifically block Myc/MAX and N-Myc/MAX interactions. In the MNA-NB mouse model, the compound can downregulate *MYC*/*MYCN* expression, induce apoptosis, and inhibit tumor growth with low toxicity (Castell et al., 2022). Yang et al. used small-molecule microarrays (SMMs) to identify a hairpin-containing G4 structure that targets the n-myc protein MY-8. MY-8 can reduce the level of N-Myc in MNA-NB cells (Yang et al., 2021). Quarfloxin and CX-5461 downregulate *MYCN* and activate p53 in NB cells through the inhibition of RNA polymerase I, leading to cell cycle arrest and apoptosis (Hald et al., 2019). Affinity proteomics and molecular docking-based studies have revealed that ceftriaxone inhibits N-Myc translation by targeting DEAD-box helicase 3 X-linked (DDX3X), thereby inducing apoptosis in *MYCN*-amplified retinoblastoma and neuroblastoma cells (Chittavanich et al., 2024). Indisulam, a splicing modulator that induces RBM39 degradation, shows strong activity in MNA-NB, including TH-*MYCN* models, and may enhance anti-GD2 immunotherapy in preclinical studies (Singh et al., 2021; Nijhuis et al., 2022).

PROTAC technology was first proposed by Sakamoto et al., in 2001 (Sakamoto et al., 2001). PROTAC usually consists of a ligand (mostly a small-molecule inhibitor) of the protein of interest and a covalently linked ligand of an E3 ubiquitin ligase (E3). E3 ubiquitin ligases recruit E3 to ubiquitinate and degrade the target protein (Zou et al., 2019). Currently, the PROTAC drug Vepdegestrant, which targets estrogen receptors in breast cancer, has completed phase III clinical trials (Campone et al., 2025), and PROTAC drugs such as BGB-16673, which target BTK in chronic lymphocytic leukemia, have entered phase III clinical trials (Li Z. et al., 2025). PROTAC drugs have become a research hotspot for targeting cancer-related proteins that cannot be drugged by traditional drug development methods (Li and Song, 2020; Wang et al., 2020). There are currently no PROTAC small molecules that directly target MYC family proteins. However, PROTAC molecules for BRD4 and Aurora A, which are closely related to *MYCN*, have been studied.

In addition to conventional small-molecule inhibitors, PROTAC technology offers a promising approach for targeting the AURKA–N-Myc axis. Recently developed AURKA-directed PROTACs have shown potent ability to degrade AURKA (Adhikari et al., 2020; Liu F. et al., 2022), disrupt its interaction with N-Myc, and reduced Aurora-A and N-Myc levels in MNA-NB cells and xenograft NB model (Nelson et al., 2025; Tang et al., 2025). These findings highlight the translational potential of AURKA PROTACs as next-generation therapeutics for MNA-NB, warranting further preclinical development and early-phase clinical trials.

The PROTAC molecule dBET57, which targets BRD4, degrades the BET protein family and *MYCN* through CRBN-mediated ubiquitination, effectively inhibiting the *MYCN* SE regulatory genes *TBX3* and *ZMYND8* in the MNA-NB xenograft model and showing therapeutic potential (Jia et al., 2022). Zhang et al. reported that MZ1 is also a PROTAC inducer that targets BET. MZ1 can inhibit MNA-NB cell proliferation and the normal cell cycle and promote apoptosis (Zhang et al., 2022). Another BET-targeting PROTAC inducer, ARV-825, effectively reduces the expression of the BET protein, thereby inhibiting the expression of *MYCN* and suppressing tumor growth in PDX mice (Li et al., 2020).

Although significant progress has been made in the fields of ubiquitination, epigenetic regulation, and small-molecule inhibitors that target *MYCN*, the treatment of MNA-NB still faces challenges in terms of drug resistance and targeting efficiency. Small-molecule inhibitors targeting the N-Myc complex provide new ideas at the level of transcriptional regulation, and the emerging PROTAC technology has shown breakthrough therapeutic potential through the selective degradation of key *MYCN* cofactors. Future research should focus on the following directions: optimizing the tumor-targeting ability of PROTACs to reduce off-target effects; combining the targeting of *MYCN* upstream and downstream pathways to overcome drug resistance; and exploring specific binding sites of the dynamic structure of the N-Myc and developing more selective inhibitors. The integration of multimodal strategies may provide new directions for the precision treatment of MNA-NB.

## Glossary

**AhR**: Aryl hydrocarbon receptor
**ALDH18A1**: Aldehyde dehydrogenase family 18 member A1
**ANM**: Multifunctional nanomedicine
**ATP13A3**: ATPase 13A3
**BET**: Bromodomain and Extra-Terminal
**BHLH**: Basic helix–loop–helix
**BORIS**: Brother of Regulator of Imprinted Sites
**BPTF**: Bromodomain PHD Finger Transcription Factor
**BSO**: Buthionine sulfoximine
**CAMKV**: Calmodulin kinase-like vesicle-associated gene
**CAR-T cell**: Chimeric antigen receptor T cell
**CRC**: Core regulatory circuitry
**CRT**: Calreticulin
**DDX3X**: DEAD-box helicase 3 X-linked
**DFMO**: Difluoromethylornithine
**DHODH**: Dihydroorotate dehydrogenase
**DKK1**: Dickkopf-1
**DKK3**: Dickkopf-3
**DSBs**: DNA double-strand breaks
**EED**: Embryonic ectoderm development
**EHMT**: Euchromatic histone-lysine methyltransferases
**EIF4EBP1**: Eukaryotic translation initiation factor 4E-binding protein 1
**ELOVL2**: ELOVL fatty acid elongase 2 gene
**ESR1**: Estrogen receptor α
**FATP2**: Fatty acid transport protein 2
**GA**: Gambogic acid
**GD2**: Disialoganglioside GD2
**GLDC**: Glycine decarboxylase
**GPX4**: Glutathione peroxidase 4
**GRHL1**: Grainyhead-like 1 gene
**GSH**: Glutathione
**HDAC**: Histone deacetylase
**HHT**: Homoharringtonine
**HIF1A**: Hypoxia-inducible factor 1 alpha
**HSP70**: Heat shock protein 70
**IDO1**: Indoleamine 2,3-dioxygenase 1
**KLHL37**: Kelch-like protein 37
**lncRNA**: Long noncoding RNA
**MCTs**: Monocarboxylate transporters
**MIAT**: Myocardial infarction-associated transcript
miRNA: MicroRNA
**MNA-NB**: *MYCN*-amplified neuroblastoma
**MSR**: Mitochondrial stress response
**MTHFD1**: Methylenetetrahydrofolate dehydrogenase 1
**MTX**: Methotrexate
***MYCN***: V-myc avian myelocytomatosis viral oncogene neuroblastoma-derived homolog
**NB**: Neuroblastoma
**NEN**: Niclosamide ethanolamine
**NGF**: Nerve growth factor
**NK cell**: Natural killer cell
**OS**: Overall survival
**PA2G4**: Proliferation-associated protein 2G4
**PDX model**: Patient-derived xenograft model
**PHGDH**: Phosphoglycerate dehydrogenase
**PIP**: Pyrrole–imidazole polyamide
**PLK1**: Polo-like kinase-1
**PP2A**: Protein phosphatase 2A
**PRC1**: Polycomb repressive complex 1
**PRC2**: Polycomb repressive complex 2
**PRDX6**: Peroxiredoxin 6
**PRMT1**: Protein arginine methyltransferase 1
**PRMT5**: Protein arginine methyltransferase 5
**RA**: Retinoic acid
**REP-204**: Erythrocyte extracellular vesicles
**ROS**: Reactive oxygen species
**RTK**: Receptor tyrosine kinase
**SAGA complex**: Spt–Ada–Gcn5-acetyltransferase complex
**SAHA**: Suberoylanilide hydroxamic acid
**SCLY**: Selenocysteine lyase
**SE**: Super enhancer
**SELENOP**: Selenoprotein P
**SEPHS2**: Selenophosphate synthase 2
**siRNA**: Small interfering RNA
**SMMs**: Small-molecule microarrays
**TAD**: Transcription activation domain
**TFAP4**: Transcription factor activator protein 4
**TfR1/CD71**: Transferrin receptor 1
**TGF-β**: Transforming growth factor β
**TLR**: Toll-like receptor
**USP7/HAUSP**: Ubiquitin-specific protease 7
**VIP**: Vasoactive intestinal peptide
